# Extract of a Letter from Mr. Philip Werner, Surgeon of the Royal Navy, and of the British Factory at Algiers, to Dr. Simmons, Containing Some Account of the Inoculation of the Small Pox in Algiers; Together with Other Miscellaneous Observations

**Published:** 1790

**Authors:** 


V. *Extract of a Letter from Mr.* Philip Werner, *Surgeon of the Royal Navy, and of the Britiſh Factory at Algiers, to Dr.* Simmons, *containing ſome Account of the Inoculation of the Small Pox in Algiers; together with other miſcellaneous Obſervations.*

I HAVE it from very good authority, both from Franks or Europeans, and from the natives of Algiers, that the inoculation of the ſmall pox has been practiſed in that country for many centuries paſt. At preſent, however, it is but little in vogue in the capital, on account of ſeveral children having died by it.

They

They have two ways of inoculating, viz. by buying* or by begging the ſmall pox from thoſe who are affected with it.

The firſt way is this: the parents, whoſe child is to be inoculated, ſend a perſon into a

* Mr. Bruce, in his account of Sennaar, the capital of Nubia, relates, that "the women, both blacks and Arabs, "thoſe of the former that live in plains, like the Shillook, "or inhabitants of El-aice, thoſe of the Nuba and Guba, "that live in mountains, all the various ſpecies of ſlaves that "come from Dyre and Tegla, from time immemorial, have "known a ſpecies of inoculation, which they call *Tiſhteree el "Jidderee*, or the buying of the ſmall pox. The women "are the conductors of this operation in the faireſt and drieſt "ſeaſon of the year, but never at other times. Upon the "firſt hearing of the ſmall pox any where, theſe people go to "the infected place, and, wrapping a fillet of cotton cloth "about the arm of the perſon infected, they let it remain "there till they bargain with the mother how many ſhe is to "ſell them. It is neceſſary that the terms be diſcuſſed ju-"daically, and that the bargain be not made colluſively or "gratuitouſly, but that one piece of ſilver, or more, be paid "for the number. This being concluded, they go home, "and tie the fillet about their own child's arm; certain, as "they ſay, from long experience, that the child infected is to "do well, and not to have more than the number of puſtules "that were agreed and paid for."—Travels to diſcover the Source of the Nile; by James Bruce, of Kinnaird, Eſq. Vol. IV. page 484.—Editor.

houſe houſe where there is a good kind of ſmall pox, to beg the favour of them to ſell them a ripe puſtule juſt ready to fall off. The price uſually paid for this is a maroon, (about three half-pence) and they immediately tie the puſtule, thus purchaſed, upon their child's arm, which they have taken care previouſly to ſcratch with a pin or needle.

The ſecond way, or that of begging the ſmall pox, as it is called, is as follows: — The child intended to be inoculated is ſent for a whole day into an infected houſe, where it takes the ſick child (whoſe puſtules are on the turn) by the hand, and begs of it to give him as good a ſort of ſmall pox as its own.

In either of theſe ways infection is generally communicated; but in caſe it is not, they never repeat the experiment, being perſuaded, when this happens, that the child is not to have the diſeaſe.

Unfortunately for thoſe who are infected, the Algerines continue to follow the method (firſt introduced and ſtill adhered to by the Spaniſh practitioners at Algiers) of keeping the patients cloſely confined in a heated room, covered with many bed cloaths, and with their own cloaths on, for theſe are never allowed to be changed till the puſtules have entirely dried up and fallen off, by which means they loſe a great many of their children.

The Cobails, or inhabitants of the mountains, have a quite different method from the two I have mentioned; for they take a needle and thread, and paſs them firſt through a ripe puſtule, and then through the ſkin between the fingers of the child to be inoculated, where the thread is left, tied in a bow knot.

The operation is made in two places, by way of ſecurity in caſe one of them ſhould fail.

Theſe people allow the patients to walk about, if they are able, during the whole courſe of the diſeaſe; and neither have recourſe to any internal remedies, nor confine them to any particular regimen, but ſuffer them to eat and drink as uſual.

In reply to the inquiry you have been pleaſed to make concerning the occurrence of hydrophobia in Algiers, I will beg leave to obſerve to you, that it is certainly a very rare diſeaſe in that kingdom; yet I am credibly informed, that, in the year 1783, a dog, with all the ſymptoms of it, bit two of the ſervants at the

Dutch

Dutch Conful (Mr. Reifs)'s country houfe, and was killed immediately afterwards to prevent any further mifchief.

Both thefe men were conveyed to the Slaves' Hofpital, the Spanifh furgeon of which fcarified the wounds pretty deeply, and then cupped them; after which he rubbed in a large quantity of mercurial ointment upon the legs that had been bit, and repeated this application every day. One of the two patients, a lad, eighteen years old, was carried off by a violent falivation on the fifth day, his tongue and throat having fwelled fo much as to occafion fuffocation. The other got well, and has had no complaint fince.

During a refidence of fix years at Algiers I have not met with any accident of this kind myfelf; but feveral perfons, on whofe authority I think I can rely, have affured me that they have heard and known of inftances of it in different parts of the country.

In the courfe of my practice at Algiers I have often been ftruck with the great frequency of impotency among the inhabitants, who folicit medical affiftance for this more than for any any other complaint. The chief caufe of it is undoubtedly to be fought for in polygamy, and in their having commerce with women very early in life. Their frequent ufe of the hot bath, to which they have recourfe not only from motives of cleanlinefs and luxury, but alfo from motives of religion on many occafions, may likewife, perhaps, have fome fhare in bringing on this complaint. The generality of the patients who have applied to me for relief in fuch cafes, inftead of being exhaufted or emaciated, as you might perhaps expect, were, on the contrary, fat, and apparently healthy.

In thefe cafes they make ufe of the hotteft fpices, and likewife of mufk, amber, and zibeth, as remedies which they fuppofe to poffefs the greateft efficacy.

The Turks and Moors, in general, have an idea that camphor tends to bring on this complaint, and for this reafon object to taking it as a remedy in other difeafes.

